# Sterile Insect Technique in an Integrated Vector Management Program against Tiger Mosquito *Aedes albopictus* in the Valencia Region (Spain): Operating Procedures and Quality Control Parameters

**DOI:** 10.3390/insects12030272

**Published:** 2021-03-23

**Authors:** Carlos Tur, David Almenar, Sandra Benlloch-Navarro, Rafael Argilés-Herrero, Mario Zacarés, Vicente Dalmau, Ignacio Pla

**Affiliations:** 1 Empresa de Transformación Agraria S.A., S.M.E, M.P. (TRAGSA), Avenida de la Industria 26, 46980 Paterna, Spain; dalmenar@tragsa.es (D.A.); sbenlloc@tragsa.es (S.B.-N.); ipla@tragsa.es (I.P.); 2 Escuela de Doctorado, Universidad Católica de Valencia San Vicente Mártir, 46001 Valencia, Spain; 3 Insect Pest Control Section, Joint FAO/IAEA Division of Nuclear Techniques in Food and Agriculture, Wagramerstrasse 5, P.O. Box 100, A-1400 Vienna, Austria; rargiles@hotmail.com; 4 Departamento de Ciencias Básicas y Transversales, Facultad de Veterinaria y Ciencias Experimentales, Universidad Católica de Valencia San Vicente Mártir, C/Guillem de Castro 94, 46001 Valencia, Spain; mario.zacares@ucv.es; 5 Conselleria de Agricultura, Desarrollo Rural, Emergencia Climática y Transición Ecológica, Ctra Alicante-Valencia s/n Apdo correos 125, 46460 Silla, Spain; dalmau_vic@gva.es

**Keywords:** SIT, vector control, insect production, mass rearing, dengue, Europe

## Abstract

**Simple Summary:**

The Asian tiger mosquito *Aedes albopictus* (Skuse, 1894) is an invasive species responsible for the transmission of arboviruses such as dengue, Zika and chikungunya. The rapid expansion of this species globally is the result of a lack of effective control methods. In this context, the sterile insect technique (SIT) is an emerging tool for controlling mosquito populations. The Agriculture Department of the Valencian Region (Spain) is promoting a pilot project to evaluate the efficacy of the sterile insect technique as part of an integrated vector management program against *A. albopictus*. From 2018 to 2020, sterile male releases were carried out in two pilot sites, releasing more than 15 million sterile males over 80 ha. The present work describes the laboratory studies carried out to evaluate the performance of irradiated males to assess the feasibility of the SIT before release in the field, as well as the production and quality control parameters obtained in rearing activities. The obtained values in terms of production and quality control and the proposed rearing methodology can be useful for designing a medium-scale mosquito-rearing pipeline.

**Abstract:**

*Aedes albopictus* and *Aedes aegypti* are the main vectors of arboviral diseases such as dengue, Zika and chikungunya viruses. About a third of the world population is currently at risk of contracting *Aedes*-borne epidemics. In recent years, *A. albopictus* has drastically increased its distribution in many countries. In the absence of efficient mosquito vector control methods, the sterile insect technique (SIT) is presented as a very promising and environment-friendly control tool. The Agriculture Department of the Valencian Region is promoting an ongoing pilot project to evaluate the efficacy of an integrated vector management program (IVM) based on the use of the SIT as the main method of control. The laboratory studies for evaluating the entomological efficacy of SIT through the phased conditional testing process recommended by World Health Organization and the International Atomic Energy Agency (WHO-IAEA) are addressed. This study describes the routine operating procedures and quality control parameters for the medium-scale rearing of sterile male *A. albopictus*. More than 15 million sterile males have been produced and released in an area of 80 ha between 2018 and 2020. Of the initial L1 larvae, we recovered 17.2% of male pupae after sex sorting to be sterilized and released on the field, while the rest of the pupae remained available to maintain the rearing colony. The residual percentage of females after sex sorting was on average 0.17%. The obtained values in terms of production and quality control as well as the proposed rearing methodology can be useful for designing a medium-scale mosquito-rearing pipeline.

## 1. Introduction

The Asian tiger mosquito *Aedes albopictus* (Skuse, 1894) is an invasive species responsible for the transmission of arboviruses such as dengue, Zika and chikungunya [1]. In recent years, the distribution of *A. albopictus* and *Aedes aegypti* has increased drastically in all continents, and consequently, these arboviral diseases have become a global health concern [2]. *Aedes albopictus* was first detected in Spain (Catalonia) in 2004 [3], and since then, it has spread in all Mediterranean regions of Spain and in Basque Country [4]. In 2018, the first autochthonous cases of dengue were detected in Spain, which provided evidence of *A. albopictus* being an effective vector of this virus in continental Europe [5,6,7]. In the absence of efficient vaccines to prevent these diseases, vector control remains a key strategy. Over several decades, the fight against vector-borne human diseases has been based on the use of insecticides. However, the resistance of mosquitoes against these chemicals has been widely reported, and few insecticides are currently approved for public health use [8]. Consequently, more sustainable and environment-friendly vector control tools are required. The sterile insect technique (SIT) is a birth control method which consists of the production and release of sterile males to mate with wild females in the field. As a consequence, sterility is induced in the native wild female population, which will decline over the generations [9].

First studies on the application of SIT against mosquitoes started in the 1960s. In 1967, an isolated population of *Culex quinquefasciatus* was successfully eliminated in Myanmar by releasing mosquitoes which were sterile due to *Wolbachia*-induced cytoplasmic incompatibility [10]. The release of chemosterilized males resulted in suppression and elimination of *Culex quinquefasciatus* on an island of Florida in 1969 [11]. The same strategy was deployed in El Salvador, achieving a 99% reduction in *Anopheles albimanus* wild population [12]. Interest in this technique was only recently revived and focused on its potential as a tool for the population suppression of *Aedes* mosquitoes. Several SIT trials have been initiated in different regions of the world to fight against mosquito vectors and they are currently in their initial phase [13]. Their successful application depends on many parameters including the ability of mass-rearing facilities to produce sufficient numbers of sterile males [9,14]. The SIT-incompatible insect technique (IIT) program in Guangzhou of China is currently the largest release program, producing and releasing more than 160,000 *Wolbachia*-infected and irradiated male *A. albopictus* per hectare each week in 2016 and 2017 [15].

In Europe, the first pilot field trials of SIT have been performed in three villages of North Italy, releasing *A. albopictus* males sterilized by gamma radiation. The sterility level in the wild population reached 70–80%, followed by a similar reduction in the egg density recorded in the ovitraps [16].

The increasing interest in the application of SIT against mosquitoes is accelerating the development of equipment, guidelines and operational protocols for rearing, sex sorting, irradiation, transport and release of sterile male mosquitoes. There is considerable literature available in respect to methods and devices for mass-rearing at different stages [17,18,19,20,21,22,23]. However, specific information on the different production strategies implemented in the ongoing programs is missing. In addition, in view of several ongoing and planned SIT field trials against *Aedes* mosquitoes, there is a lack of referenced and standardized mass-rearing methodology which would support new programs.

The Agriculture Department of the Valencian Region has been promoting a pilot project to evaluate the efficacy of an integrated vector management program (IVM) based on the use of the SIT as the main method of control. The project roadmap has been based on the phased conditional approach framework proposed by World Health Organization and the International Atomic Energy Agency (WHO-IAEA) for the implementation of SIT programs [13]. Phase I described in these guidelines focuses on the definition of laboratory studies to assess the feasibility of the SIT when it will be applied on the field. On the other hand, phase II involves semi-field and field trials in order to adjust the technique before scaling it up to larger areas. Both phases have been fulfilled in this project over four years.

The present study reports the laboratory studies performed in phase I for evaluating the entomological efficacy of SIT. Moreover, the different steps of the mass-rearing process and quality control protocols for sterile male *A. albopictus* in support of the SIT program in the Valencia Region are detailed. The key parameters that describe the production process are analyzed to evaluate its efficiency. The results will allow comparison of the described process with those followed in different programs and will contribute to the development of a standardized procedure for the mass-rearing of *Aedes* mosquitoes for SIT applications.

## 2. Materials and Methods

### 2.1. Risk Assessment

A risk assessment including human health and ecological aspects was addressed in phase I. All activities including male production and irradiation were approved by the Regional Government, which has the regulatory authority in Spain, and were deemed in accordance with the national law for occupational risk prevention and national legislation on invasive species.

### 2.2. Rearing Colony

**Figure 1:**
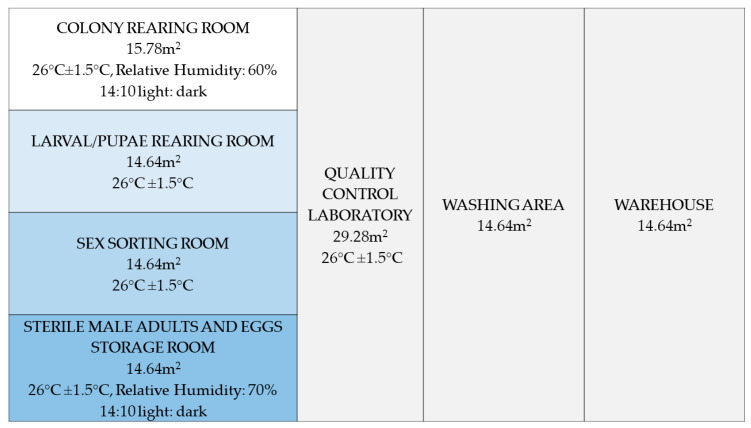
Layout of the Valencia medium-scale rearing facility.

A mosquito colony was initiated from eggs collected in different municipalities of the Castellon and Valencia regions in 2014. The strain was maintained using standard procedures [24] until 2017, when the colony was expanded prior to the implementation of the pilot project. The mass-rearing facility was divided into seven areas as shown in Figure 1.

**Figure 2:**
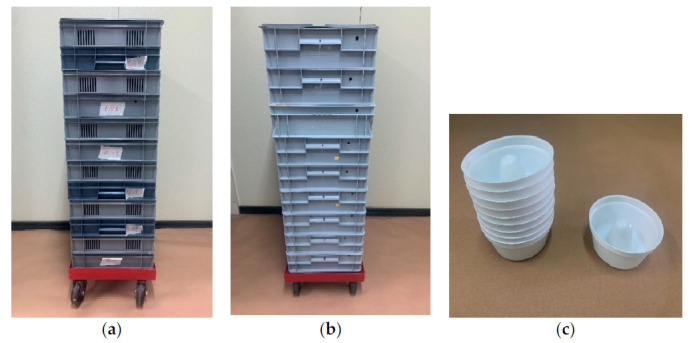
(**a**) Egg maturation trays. (**b**) Larval rearing trays. (**c**) Irradiation cups.

The size of the rearing facility was estimated for a maximum production of 300,000 sterile males per week. The colony rearing room was equipped with a double bio-security door and air curtain to prevent mosquitoes escaping. Adults were reared in 40 × 40 × 40-cm methacrylate cages that were stored on stainless steel shelves. Each methacrylate cage was filled with 10,000 immature mosquitoes (L4 and pupal stages) from the leftover of sex-sorting operation at a female:male ratio of approximately 3:1. The number of cages maintained depended on the production objectives. As an example, 18 adult cages were set up weekly for a production of 240,000 sterile males. Adults were fed with a pasty sucrose-based food originally used to feed bees (Apicomin Jarabe Denso, Kessler Iberica, Montserrat, Spain). This food was applied by sticking 10 g on the cage wall and it was renewed twice a week. Females were offered a blood meal daily from day 6 to day 17 after the loading of the cage. Defibrinated fresh pig blood was obtained from an authorized slaughterhouse according to EU rules twice a week and 150 mL per cage was offered daily to the females inside a collagen sausage (FIBRAN S.A., Girona, Spain) heated at 38–39 °C. Adult cages were maintained for 21 days. From day 12 after cage loading, eggs were collected on filter papers every 3–4 days and stored in 20 40 × 60 × 7-cm plastic trays stacked on a base with wheels. Four days later, reticulated trays were inserted between the trays to facilitate aeration and prevent the appearance of mold (Figure 2).

Five days a week, 400,000 to 450,000 eggs were transferred to 1000-mL sealed containers with nutrient broth solution at up to 17,000 eggs per jar [21]. Hatched L1 larvae were dosed using a mosquito larval counter (Radiation General Ltd., Budapest, Hungary) [19]. The presence of debris causes an overestimation of the number of larvae. In order to improve the efficiency of the larval counter, the larvae were placed at the end of a 40 × 60-cm tray with a light source to stimulate the larvae to actively swim towards the dark end of the tray, due to their negative phototactic behavior. In this way, the larvae were separated from the eggshells. Larvae were reared for six days in 40 × 60 × 12-cm plastic trays stacked with 10,000 larvae and 5 L of water per tray. Larvae were fed with the IAEA diet based on liver powder, tuna meal and brewer’s yeast [25]. Eight days after hatching, the pupae were collected for sex sorting. The sex sorting was performed using a plate separator [26]. After separating male pupae, the remaining larvae and pupae were returned to the trays and used the next day to load the rearing cages.

A ^60^Co Gammacell 220 irradiator, located in the *Ceratitis capitata* mass-rearing facility in Caudete de Las Fuentes (Valencia), was used to sterilize the male pupae. The dose rate of the ^60^Co Gammacell 220 irradiator was estimated from 2430 Gy/h in January 2018 to 1656 Gy/h at the end of November 2020. Consequently, the irradiation time was increased to adjust the required dose. A dosimetry system was established to monitor the dose rate of the irradiation. A Gafchromic MD-V3 Dosimetric Film (Ashland, Bridgewater, NJ, USA) with an Optical Density Reader (DoseReader4, Radiation General Ltd., Budapest, Hungary) was used according to the Insect Pest Control Laboratory-IAEA SIT dosimetry protocol [27]. Male pupae were transferred to irradiation cups 24 h after sex sorting. Each cup contained 750 males, estimated volumetrically using a tube with a perforated base and graduated/calibrated to 750 pupae. Irradiation cups were specifically designed for the procedure and consisted of stackable plastic containers with a ring-shaped cavity, with inner and external radii of 2 and 4 cm. This design ensured that when the cups are stacked for insertion into the canister of the irradiator, there is a 1-cm chamber at the base of each cup where the pupae are located in water. In this way, there was a balance between pupae load and dose homogeneity, taking into account the dose mapping of the Gammacell 220 irradiator.

The pupae were transported by car from the rearing facility (ca. 1 h) to the irradiator facility in these cups filled with water up to 0.5 cm. For transportation, cups were placed in 40 × 60 × 12-cm plastic trays. Eight cups were irradiated per cycle. Each stack was irradiated centered in the X, Z and Y axes of the cylindrical chamber of the Gammacell irradiator by using a holder, at the desired dose of 48 Gy. Thereafter, each cup was introduced directly into a release cage, which were 17 × 18 × 28-cm plastic containers with two sides of plastic mesh. A plastic cup with a 10% sucrose solution was placed inside each release cage. After three days, the irradiation cups were removed.

### 2.3. Laboratory Studies for Evaluating the Entomological Efficacy of SIT Based on WHO-IAEA Phase Conditional Approach

**Figure 3:**
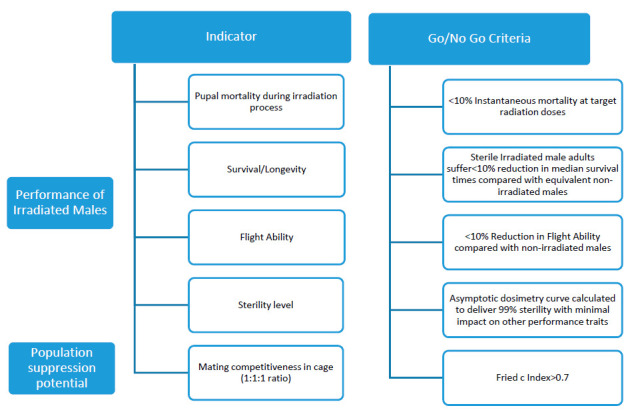
Laboratory studies for evaluating the entomological efficacy of the sterile insect technique (SIT) through the phased conditional testing process in line with the World Health Organization and the International Atomic Energy Agency “Guidance Framework for Testing the Sterile Insect Technique as a Vector Control Tool against *Aedes*-Borne Diseases”.

To evaluate the quality of the produced sterile males before release in the field, laboratory studies were carried out in line with the WHO-IAEA “Guidance Framework for Testing the Sterile Insect Technique as a Vector Control Tool against *Aedes*-Borne Diseases” [13] (Figure 3).

- Determination of irradiation dose. The target irradiation dose for the male pupae was established as the dose required to obtain 99% sterility in males. To estimate the target dose, six batches of 75 pupae that were 24–36 h old were irradiated with different doses (0, 20, 40, 50, 60 and 80 Gy) in a ring-shaped container with inner and outer radii of 2 and 4 cm, placed in the geometrical center of the irradiation chamber. A sample of 50 pupae per batch was selected to emerge in a 24.5 × 24.5 × 24.5-cm BugDorm-4S2222 insect rearing cages (MegaView Science Co., Taichung, Taiwan) and males were allowed to mate with 50 virgin females for 5 days. After three days of blood feeding, the females were isolated in *Drosophila* vials that contained water and filter paper to stimulate oviposition. The eggs oviposited by each female were counted, dried for four days and allowed to hatch for 48 h by submerging the filter papers in a solution of nutrient broth. The ratio of hatched eggs over the total was estimated for each female.
- Survival. Two batches of irradiated male pupae were selected for the survival analysis. A sample of 50 pupae of each batch was allowed to emerge in BugDorm-4S2222 insect rearing cages (MegaView Science Co., Taichung, Taiwan) with access to a 10% sucrose solution. Simultaneously, 50 non-irradiated male pupae (control) were allowed to emerge under the same conditions. The dead males were counted daily in the following 20 days.
- Instant pupal mortality during irradiation. The number of dead pupae after irradiation was counted in four irradiation containers for 3 days (n = 12). Before introduction in the irradiator, the dead pupae were removed, and the dead pupae immediately after irradiation were counted.
- Pupal emergence and flight ability. A flight organ device was used to estimate both pupal emergence and flight ability [28]. Each flight organ was composed of 120 tubes (diameter, 10.1 mm; length, 400 mm). Eight paired simultaneous tests were conducted (control vs. irradiated).
- Mating competitiveness. BugDorm-4S2222 insect rearing cages (MegaView Science Co., Taichung, Taiwan) were filled with different ratios of untreated and irradiated male mosquitoes to estimate the competitiveness of irradiated males. Three cages were used with 30 individuals of each category: sterile males, non-irradiated males and virgin females. Three additional cages with 30 individuals per category contained only sterile males and females and another three contained only non-irradiated males and females. After 5 days, three blood meals were offered to the females, and an oviposition cup with filter paper and water was introduced into each cage. The number of eggs was counted for each cage, and the eggs were allowed to hatch in a solution of nutrient broth for 24 h. The ratio of L1 larvae versus the number of eggs was used as a hatching rate index. The hatching rates of the different mosquito combinations were used to estimate the Fried competitiveness value c [29].

### 2.4. Rearing Parameters and Routine Quality Control Measures

In recent years, rearing parameters and protocols have been developed to evaluate the quality and efficiency of mosquito sterile male production [30,31,32,33]. However, there are no guidelines with respect to reference parameters as in fruit fly mass-rearing [34]. In order to know the status of the colony and to estimate available production, routine production and quality control parameters have been established. These are:

- Adult colony rearing
  - Survival rate in the adult rearing cages. To estimate the survival of individuals, a triangular, transparent 16 × 16 × 22.6-cm Plexiglas sheet was placed in the upper right corner of a randomly selected cage assembled each day. Every day at the same time, during the lifetime of the cage, the number of resting male and female mosquitoes was estimated as a relative measure of the survival of the mosquitoes.
  - Egg production and female fecundity. The number of eggs on each oviposition filter paper was visually estimated by an expert technician by comparison with a set of reference oviposition filter papers for which the number of eggs had been estimated [35]. For each oviposition paper, female fecundity was estimated as the egg number divided by the expected number of active females. The expected number of females for a given collection day was approximated using the female survival model. The accumulated fecundity for each cage was estimated as the sum of fecundities of their collected oviposition papers.
- Larval rearing
  - Hatching rate. A sample of 2 to 6 oviposition papers was randomly selected after their use in the hatching jars. A random sample of about 200 eggs along the surface of each paper was selected, and the number of hatched and unhatched eggs was counted under a stereomicroscope.
  - Pupation at sex sorting and sterile male pupae production. Eight days after eggs hatching, pupae were sexed. Prior to sex sorting, a larval rearing tray was chosen at random and used as a sample for the estimation of pupation parameters. The sample trays were sorted and counted simultaneously with the sex sorting of the production trays. The number of individuals in the three categories of the output (male pupae, female pupae and larvae) was estimated volumetrically. Three tubes (one for each category) were specifically designed for this purpose with a perforated base and graduated/calibrated to 100 individuals. The percentage survival at sex sorting was estimated as the sum of three categories divided by 10,000. The pupation rate at sex sorting was estimated as the sum of male and female pupae divided by the total number of individuals. The percentage of male pupae was estimated as the number of male pupae divided by the total number of pupae at sex sorting. Concerning the total production of male pupae to be sterilized, it was obtained from volumetric dosing into cups for irradiation.
- Sex sorting
  - Residual female contamination. The sex sorting of 240,000 males per week involved two technicians five days a week working eight-hour days. As the residual contamination of females in sex sorting depends in part on the skill of the technician, from each of the release batches, 5 cages from each technician were randomly selected on the third day after irradiation and visually checked for females. All females found in the release batches were removed by means of a manual aspirator. A total of 830 batches were visually inspected for the presence of females after the emergence of adults. If the average residual percentage of females for the technician exceeded 1%, 5 more cages were randomly selected. If this percentage was maintained at a level higher than 1%, the entire batch was checked and females were removed before leaving for the field.

### 2.5. Statistical Analysis

All statistical analyses were carried out using R [36]. The survival rate was approximated as the slope of a fitted density–time model. A Poisson Linear Mixed-Effect Model using the lme4 package [37] was used with the resting number as the dependent variable, the day (starting on the fourth day after pupal introduction, day with maximal counts) as a fixed-effect term and the cages as a random effect term. The exponentiated slope of the model was used as an estimate of daily survival. The half-life (day with resting density = 50% of the peak density) was also derived from the model.

The female fecundity and the total number of eggs per cage were log-transformed to analyze the effect of time on egg production. A Gaussian linear mixed effects model with the number of days from the introduction of pupae in the cage (days) as a fixed effect term and the cages as a random effect term was fit to the data, using the lme4 package [37].

The egg hatching rate decay with storage time was analyzed by means of a binomial linear mixed effect model using the lme4 package [37] considering the number of hatched/not hatched eggs as the dependent variable, the number of weeks from collection to hatch as a fixed effects term and the oviposition papers as a random effect term. The hatching data for 555 oviposition papers were included in the analysis.

A binary general linear model was fit to estimate the effect of irradiation on pupal emergence and flight ability parameters, measured in the flight organ. Success/failure in the parameters was used as dependent variable and treatment and test number as independent variables.

A binary general linear model was fit to estimate the relationship between hatching success and irradiation dose. The fit model was used to estimate the target irradiation dose that obtains 99% sterility in male pupae.

The effect of irradiation over survival was analyzed by fitting a Cox proportional hazards model with treatment as an independent variable. Males that died, escaped or survived the entire trial were considered censored. The survival R package was used for the analysis [38].

## 3. Results

### 3.1. Laboratory Studies

All the indicators for phase I described in the Guidance Framework for Testing the Sterile Insect Technique as a Vector Control Tool against *Aedes*-Borne Diseases were satisfactorily fulfilled.

**Figure 4:**
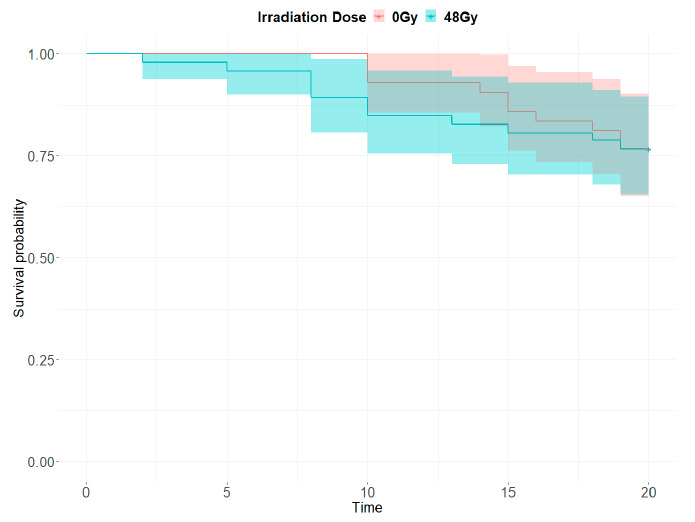
Survival plots of irradiated (48 Gy) and untreated (0 Gy) male *Aedes albopictus* over 20 days.

- Determination of irradiation dose. The parameters for the fit model are shown in Table 1. According to the table, the predicted target dose was 48 Gy. Its associated hatching rate was 0.88%, equivalent to an induced sterility (Abbot’s corrected) of 98.93% [39].
- Survival. In both control and irradiated groups, at least half of the mosquitoes survived more than 20 days. There were no significant differences in the longevity of the irradiated mosquitoes (48 Gy) compared to the control treatment (0 Gy) (hazard ratio = 1.194, z = 0.661, *p* = 0.509) (Figure 4).
- Instant pupal mortality during irradiation. On average, 0.49% of the pupae died during the irradiation process (SD = 0.27).
- Pupal emergence and flight ability. On average, 97.1% of the irradiated pupae and 97.6% of the non-irradiated emerged to adult. Differences were not statistically significant for untreated and irradiated males (estimate = −0.221, Std. Error = 0.381, z = −0.581, *p* = 0.561). On average, 85.1% of the irradiated pupae and 90.8% of the non-irradiated succeeded in the flight ability test, which was a statistically significant difference (Estimate = −0.544, SE = 0.201, z = −2.712, *p* = 0.007).
- Mating competitiveness. The Fried c value was on average 0.92.

### 3.2. Rearing Parameters and Quality Control Measures

**Figure 5:**
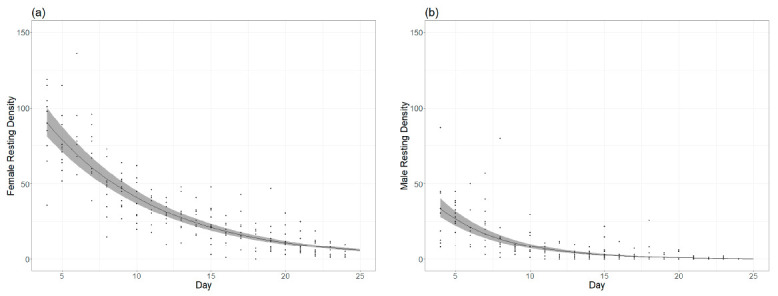
(**a**) Survival rate of females over time. (**b**) Survival rate of males per day.

- Adult colony rearing
  - Survival rate. Results are shown in Table 2 and Figure 5.

The average daily survival rate estimated for females was 87.7% and 79.7% for males. The half-life (density = 50% of the peak density) of the female population, estimated from the model, was 9.2 days from the introduction of the pupae. The half-life for the male population was 7.1 days.

**Figure 6:**
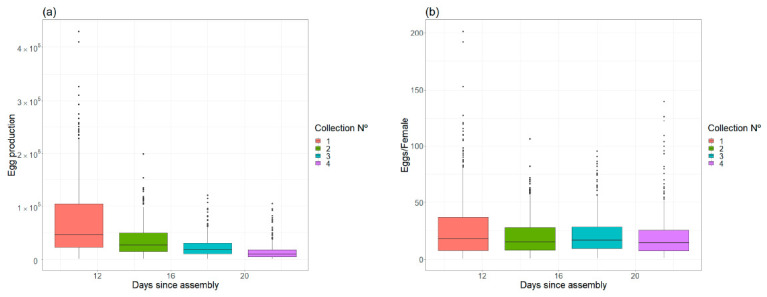
(**a**) Egg production in each egg collection. (**b**) Eggs per female over the time since cage assembly.

Egg production and female fecundity. The total egg number for 1356 oviposition papers obtained from 339 cages was analyzed (four collections per cage) to estimate female fecundity. The total egg production, production per cage and production per female pupae are shown in Table 3. Egg production in each egg collection and eggs per female over the time since cage assembly are shown in Figure 6.

**Figure 7:**
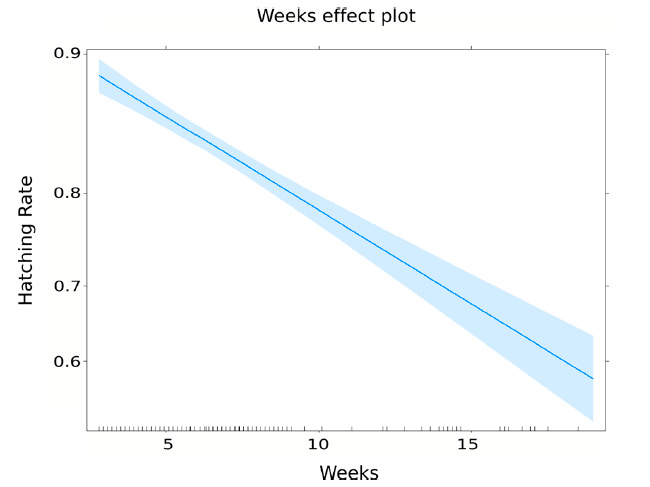
Hatching rate vs. time in storage (weeks).

The daily fecundity corrected by the female survival model was 6.7 eggs/female-day. The fecundity per collection was 23.3 eggs/female-collection (SD = 23.93). The total egg production in a cage declined with time. From the total egg production, 50.24% corresponds to the first collection, and 23.89%, 15.84% and 10.03% to the second, third and fourth collections, respectively. However, the production per female (individual fecundity) remained relatively constant. The slope of the regression for egg production over time was significantly different from 0, while the slope of the individual fecundity was not (Table 4).

ii. Larval rearing
  - Hatch rate. Results of egg hatching are shown in Table 5. According to the model, the egg hatching rate was declining with time in storage (Figure 7). After one week of collection, the expected hatching rate was 90.6% (Std. Error = 0.56%). After 5 weeks, it was reduced to 86.2% (Std. Error = 0.43%), and after 20 weeks, it dropped to 54.8% (Std. Error = 3.40%)
  - Pupation at sex sorting and male pupae production. A total of 332 trays were used as samples for the estimation of pupation parameters. On average, 0.172 male pupae were recovered at sex sorting from each L1 reared. The specific estimates for the different parameters of pupation are shown in Table 6.

**Figure 8:**
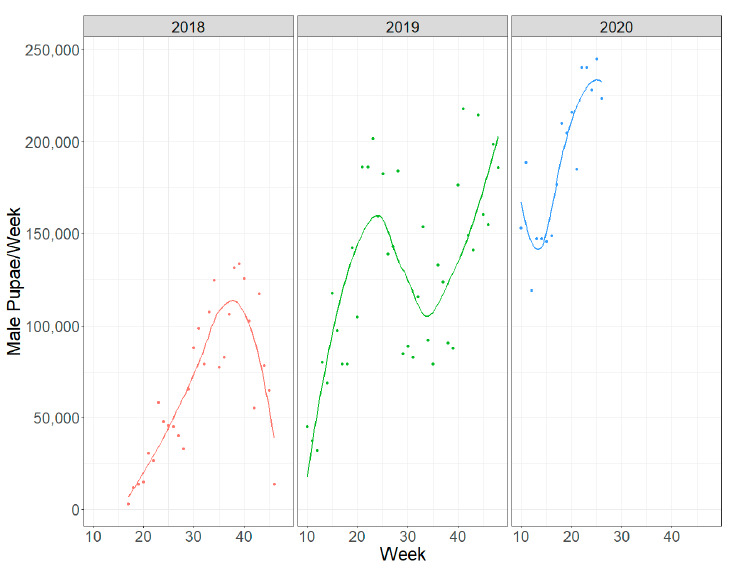
Male pupae production over the time.

The total production of male pupae fluctuated, with an increasing trend over the course of the project (Figure 8). On the submission date, the production capacity was about 240,000 male pupae/week.

iii. Sex Sorting
  - Residual female contamination. The presence of females in the release containers was on average 0.17% of the total individuals (SD = 0.24). Only 0.59% of the batches had a female contamination rate higher than 1%. We estimate that the protocol of inspection reduced the residual presence of females to 0.15%.

## 4. Discussion

The objective of the SIT pilot project against *A. albopictus* in the Valencian Region was to evaluate the use of the sterile insect technique as part of an integrated vector control program. Prior to the mass production and field release phase, the suitability of the project was evaluated at a laboratory level following the criteria proposed by WHO-IAEA [13]. All criteria in this document were met, supporting the transition to a second phase consisting of increasing production to test the release of sterile males in the selected areas. The irradiation dose of 48 Gy needed to achieve 99% sterility in males seems high compared to previous experiments [40,41,42,43]. Nonetheless, there is recent evidence that several factors such as pupae age and oxygen availability affect the response to radiation in mosquitoes [44,45]. In addition, in small self-contained gamma irradiators such as the Gammacell 220, the irradiation intensity is spatially heterogeneous and only the center of the irradiation chamber receives the dose of reference. These factors are difficult to control in mass production, and a higher dose assures that all the pupae are treated over a minimal irradiation threshold. Ensuring acceptable sterility implies a loss of quality, which is reflected in the post-emergence flight ability but not in the longevity test. Irradiation may also affect the male mating competitiveness. In our case, the mean value of Fried Index c was 0.92, which is higher than the value recommended in the WHO-IAEA “Guidance Framework for Testing the Sterile Insect Technique as a Vector Control Tool against *Aedes*-Borne Diseases” [13] (c > 0.7).

The sterile insect technique and other rear-and-release vector control methods, such as IIT or the Release of Insects carrying a Dominant Lethal (RIDL) [46], depend on the capacity to produce large quantities of insects with an adequate quality that ensures their effectiveness in the field. Therefore, the optimization of production parameters is key to obtain efficient production in a successful SIT program against vector mosquitoes. There is extensive literature describing optimized rearing methods and their outcomes in experimental contexts [20,47,48,49,50,51]. References showing real parameters of a mass production in practice are, however, scarce [16,52], as there are few projects that produce sterile males on a medium or large scale.

The final objective of adult rearing is the production of eggs, and maximization of fecundity is desirable. Research efforts have usually been focused on finding the optimal shape and vertical resting surface [47,53], sex ratio [47,54] and blood feeding methods [54,55]. In our rearing cages, egg production was highly variable (Figure 6), probably influenced by blood quality and rearing experience. The number of eggs laid per initial female pupae reached an average of 21.03. Even taking into account differences due to biotic factors such as different strains, and also due to colonization processes, we consider that this productivity is relatively low compared to laboratory studies [20,47,54,56], although the rearing methods were basically equivalent. There is no comparable information for mass-rearing programs. The chosen design of the adult cage probably did not influence the productivity. According to Balestrino et al. [53], medium-sized cubic cages, such as the ones used in our program, are similar in productivity to large space-optimized cages, and Zhang et al. [48] found that short cages allow for higher blood-feeding rates and egg production. The vertical resting surface density (1.56 mosquitoes/cm^2^) was similar to the optimal density proposed by Zhang et al. [47] (1.48 mosquitoes/cm^2^), although both are high compared to the density of 0.8 mosquitoes/cm^2^ used in similar experiments [53,54,56]. It should be noted that the results may have been affected by the method of estimating the number of produced eggs. Visual egg estimation is a fast method in the context of mass production when personnel are trained but needs to be improved to obtain a more accurate measure.

Concerning the life cycle of rearing adult cages, Zhang et al. and Maïga et al. [47,56] suggested a two-week cycle for *A. albopictus* in a medium-scale rearing facility to increase efficiency per cage. In our rearing process, 74% of the eggs were collected in the first two weeks, and 26% in the third one. Currently, the cost of dismantling, cleaning and reassembling each cage is high, and the maintenance of the cages for three weeks makes production more cost-effective.

The total egg production per cage is related to the fecundity of females but, apparently, also to the mortality rate. As described in the Results section, egg production per surviving female remained constant during the four collection dates, with female mortality likely being the factor that reduced the productivity of the cage over time. The fecundity per estimated female in the first gonotrophic cycle can be considered low (30 eggs/female) compared with laboratory studies (mean value ranging from 13 to 143 eggs per female) [53,55,57,58,59,60,61]. In any case, the methodology used to estimate the fecundity of the females was different from the referenced works, since it was obtained from eggs collected per cage and the survival curve was obtained according to the date of collection, and some artefact may be influencing the results. This methodology was used because the objective of the presented values was to evaluate the productivity of the rearing cages in a routine mass-rearing context. The survival of females was similar to that reported by Maiga et al. [56] with a comparable methodology (88.5%). In our colony, the mortality of males was higher than females. Maiga et al. reported similar decay rates between males and females with an average density, but Balestrino et al. [53] found that differences in mortality between sexes were linked to density. It is possible, then, that males are more affected by overcrowding than females.

The hatching rate of the eggs decreased over time in storage, as has been generally observed [17]. It is, therefore, desirable to define a strategy that balances the maintenance of an egg reserve and an acceptable hatching rate. A stock of eggs is advisable to compensate for unforeseen events in production. The hatching rate can be kept above 80% for nine weeks, as described in Table 5. These results are consistent with those obtained by Zheng et al. [17], which obtained a hatching rate greater than 80% after 10 weeks using nutrient broth as a hatching medium. Longer-term egg conservation is highly recommended in temperate regions with a stationary wild population dynamic. In the period when field populations are low or non-existent, it would be possible to continue the production of an egg bank, which would reinforce the high population season releases.

The goal of larval rearing is not only to provide pupae for the production of sterile adult males to be released, but also to restock the egg colony. In our program, male pupae were sex sorted on the 8th day after hatching. Of the initial L1 larvae (males and females), 17.2% of male pupae were recovered at sex sorting to be irradiated and released in the field. The rest of the pupae produced in the trays remained available to maintain the rearing colony. These values are slightly higher than the productivities of equivalent mass rearing programs with *A. albopictus* [16] or *A. aegypti* [52], but significantly lower than the values for simulations of mass-rearing obtained in laboratory experiments [48,49,50]. The use of a multi-step sex sorting method based on artificial intelligence can recover almost the totality of the male production [62].

The average contamination rate of females in the batches of the released males was 0.15% using the Fay–Morlan apparatus [26]. Carvalho et al. [52], using a similar device, released only 0.02% of females, probably related to the more efficient size separation of *A. aegypti* due its more pronounced sexual dimorphism [63]. A more sophisticated method was used by Crawford et al. to release a female every 900 million males [62]. On the other hand, Bellini et al. [16], using a less efficient separation method with *A. albopictus*, obtained 1.21% females. Compared to larval rearing, the labor invested in sex sorting is considerably high [64,65] and its optimization could boost the productivity in mosquito mass-rearing facilities. New approaches to mosquito sex sorting are being developed to develop large-scale programs against vector mosquitoes [62,63,66,67]. The parameters derived from laboratory experiments likely overestimate the productivity since they are obtained in ideal conditions, but parameters derived from a real context offer a more informative point of view of the process. The availability of realistic production parameters is useful as a reference for the design of future projects and it is necessary in order to understand and optimize the production process. Currently, there are few projects that produce sterile males of mosquitoes on a medium or larger scale that can offer this kind of information. It is also noteworthy that during the four years of the project, parameters such as the number of eggs per female or male production increased considerably, partly due to an adaptation of the colony to the rearing conditions, but also mainly due to improved procedures and training of the staff.

In addition, most of the designs and methods proposed for mosquito rearing tend to be biologically optimized (gross production per individual) rather than in terms of handling efficiency (gross production per hour of labor). Despite the fact that larger cages and larval rearing trays present a greater capacity and allow a reduction in the number of units, their handling and cleaning become more complex than small units due to bigger dimensions. In order to optimize cost efficiency in a large number of small cages, efforts are being made to automate the rearing processes. In this sense, adaptation to industrial stacking containers such as the Standard Euro Containers can allow easier adaptation of washing, storage or handling systems already existing in the industry, which are usually highly optimized due to their wide range of applications.

On the other hand, the use of self-stacking trays reduces the space required and facilitates its displacement by means of rolling bases. Likewise, the height of the tray towers can be adapted to the dimensions of the rearing chamber or the production level. The weight (5 kg) and dimensions of the trays (60 × 40 cm) allow for easy handling.

The proposed methodology allows for evaluating the efficiency of the SIT as part of an integrated vector control program, by achieving optimal sterile/wild male ratios in field trials complying with the standard quality parameters proposed by IAEA-WHO. However, a greater effort has to be made to automate the rearing, irradiation and sterile male release processes to enable scaling up to an operational level. For this purpose, the incorporation of automatic systems for feeding larvae and adults, sex sorting, methods of irradiation in adults and release with automatic terrestrial or aerial systems are expected to be implemented in the future.

## 5. Conclusions

Laboratory studies carried out before starting sterile male releases on the field following a phased conditional testing process have been described in this work. The results obtained are in line with those presented in the WHO-IAEA “Guidance Framework for Testing the Sterile Insect Technique as a Vector Control Tool against *Aedes*-Borne Diseases” and can reinforce its suitability for future SIT pilot projects.

Routine rearing parameters and quality control measures have been detailed after three years of medium-scale sterile male production. The obtained values can be useful for designing a medium-scale mosquito-rearing pipeline.

## Figures and Tables

**Table 1:** Parameter estimates for the model relating hatching rate to irradiation dose.

Egg Hatching	Estimate	Std. Error	z Value	*p*-Value
Intercept	1.694	0.033	50.40	<0.0001
Dose (Gy)	−0.133	0.001	−70.02	<0.0001

**Table 2:** Survival rate of males and females. Parameter estimates of model for the relationship between resting number of individuals over a 128-cm^2^ surface versus time.

Sex		Estimate	Std. Error	z Value	*p*-Value
Female	Intercept	4.425	0.100	44.14	<0.0001
	day	−0.226	0.005	−41.69	<0.0001
Male	Intercept	5.022	0.056	89.09	<0.0001
	day	−0.131	0.002	−61.75	<0.0001

**Table 3:** Egg production parameters for three years of mass rearing of *Aedes albopictus*.

	Average Egg Production/Month	No. Eggs/Cage		No. Eggs/Female Pupae	
Year		Average	SD	Average	SD
2018	2,661,583	60,836	55,306	8.79	7.99
2019	7,946,500	140,854	123,474	20.35	17.84
2020	8,590,583	145,603	87,262	21.03	12.61

**Table 4:** Regression parameters for egg production and fecundity over the time since cage assembly.

		Estimate	Std. Error	F-Value	*p*-Value
Egg production	Intercept	12.002	0.112	110,483.4	<0.0001
	day	−0.127	0.007	365.1	<0.0001
Fecundity	Intercept	2.782	0.099	11,227.690	<0.0001
	day	0.001	0.006	0.046	0.829

**Table 5:** Egg hatching rate.

Egg Hatching	Estimate	Std. Error	z Value	*p*-Value
Intercept	2.379	0.075	31.52	<0.0001
Weeks	−0.109	0.010	−10.74	<0.0001

**Table 6:** Parameters of pupation at sex sorting.

Parameter	Mean	SD
% Survival at sex sorting	83.56	17.87
% Pupation rate at sex sorting	29.03	11.97
% Male pupae at sex sorting	75.66	8.6
Sorted male pupae/tray	1720.18	465.23

## Data Availability

All data generated or analyzed during this study are included in this published article.
